# Current status and educational needs of early clinical exposure in Korean Medical Schools: A cross-sectional survey study

**DOI:** 10.12669/pjms.41.2.10717

**Published:** 2025-02

**Authors:** Songrim Kim, Sun Young Kyung, Kwi Hwa Park, So Jung Yune

**Affiliations:** 1 Songrim Kim, PhD. Researcher, Office of Medical Education, College of Medicine, Gachon University, Incheon, Korea; 2 Sun Young Kyung, PhD. Professor, Department of Internal Medicine, College of Medicine, Gachon University, Incheon, Korea; 3 Kwi Hwa Park, PhD. Professor, Department of Medical Education, College of Medicine, Gachon University, Incheon, Korea; 4 So Jung Yune, PhD. Professor, Department of Medical Education, School of Medicine, Pusan National University, Busan, Korea

**Keywords:** Clinical clerkship, Early clinical exposure, Education, Medical, Medical students, Needs assessment

## Abstract

**Objective:**

To examine the status of early clinical exposure (ECE) programs in Korean medical schools and to determine the educational needs for ECE in undergraduate medical education.

**Methods:**

In this cross-sectional study, 30 medical education experts and 65 professors from 30 medical schools across Korea were surveyed about the status of ECE programs in medical schools and the educational needs for ECE. This survey was conducted between January and March 2024 using Google Forms, and the collected data were analyzed using frequency analysis.

**Results:**

Out of the 30 participating medical schools, 70% had implemented ECE programs. In most schools, ECE programs were required courses and offered from the first year of pre-medical phase to the second year of medical phase. Among 95 respondents, 88.4% recognized the necessity of ECE programs. Most respondents considered “understanding the role of a doctor” an essential objective (70 out of 95, 73.7%) and outcome (73 out of 95, 76.8%) of ECE programs. Most respondents considered “observation/field trips” and “reflection journal” as essential teaching methods (69 out of 95, 72.6%) and assessment methods (68 out of 95, 71.6%) in ECE programs, respectively. Furthermore, most respondents (68 out of 95, 71.6%) considered the “lack of educational support personnel” a concern in implementing ECE programs in medical schools.

**Conclusions:**

This study sheds light on the status of ECE programs in Korean medical schools. Additionally, its results regarding the educational needs for ECE have implications for the future implementation of ECE programs in Korean medical schools.

## INTRODUCTION

The landmark Flexner report laid the foundation for modern medical education. Since its publication in 1910, medical education has comprised two years of basic medicine followed by two years of clinical medicine.^1^ However, the landscape of medical knowledge and societal expectations of doctors has evolved dramatically since the 1950s. This has led to the reorganization of medical education and the introduction of early clinical exposure (ECE), a strategy that aims to enhance learning motivation by bridging the gap between basic and clinical medicine.^2^

To determine the consensus about ECE, Dornan and Bundy (2004) defined “experience” as “authentic human contact in a social or clinical context that enhances learning of health, illness or disease, and the role of the health professional”.^3^ In other words, it allows students to contact actual patients from the basic medicine stage, thus exposing them to clinical environments early. ECE can be implemented to provide this experience through interviews with patients, observation of consultations, shadowing nurses or doctors, and visits to community medical centers. ECE is an important educational strategy in enhancing the linkage between basic and clinical sciences and achieving educational outcomes such as understanding of patients, communication skills, and professionalism.

In 1993, the UK’s General Medical Council recommended early clinical contact be incorporated into integrated courses.^4^ In 1998, the World Federation of Medical Education suggested expanding the integration of basic and clinical disciplines and encouraging students to meet patients early.^5^ Furthermore, Educating Physicians, published one hundred years after the Flexner report, also recommended the incorporation of early clinical immersion to link knowledge with clinical experience.^6^ With increasing emphasis on the need for ECE in medical education, many studies have examined ECE. These studies have been conducted mainly in North America and Europe.

In other regions, the proportion of such studies increased moderately in the 2000s.^7^ In Europe, studies have explored the status of ECE programs in medical schools.^8^,^9^ In India, ECE guidelines have been established for undergraduate medical education, and studies have analyzed the educational effects of ECE in basic medicine.^10^,^11^ In Asia, such as China and Taiwan, empirical studies have conducted on the educational effects related to cognitive and affective outcomes of ECE programs for medical students.^12^-^14^

However, in Korea, research remains scant on ECE. The existing studies have mainly explored the experience of implementing ECE programs in a single medical school.^15^,^16^ ECE plays a crucial role in basic medicine courses, as it allows medical students to be motivated and incorporate early clinical experience with basic medicine. Additionally, ECE establishes vertical integration of medical knowledge by linking basic and clinical medicine. This calls for more attention and research on ECE in the context of Korean medical education. Therefore, it is necessary to discuss the concept of ECE and the implementation of ECE programs in Korean medical education. This study aimed to understand the status of ECE programs in Korean medical schools and determine future directions by investigating educational needs for ECE.

## METHODS

This was a cross-sectional study, with an online survey conducted at medical schools across Korea. Data were collected using an online survey conducted between January and March 2024 that targeted medical schools across Korea. Created using Google Forms (Google LLC; Mountain View, CA, USA), the questionnaire was structured to gather data on the status of ECE programs and the educational needs for ECE. Two questionnaires were prepared for the survey: one to assess the status of ECE programs and the other to determine the educational needs for ECE. Medical education experts were asked to fill out the questionnaires on the status of ECE programs and the educational needs for ECE, while professors were asked to fill out the questionnaire on educational needs. All responses were collected online and used for analysis. The survey targeted medical education experts and professors from 40 medical schools including public and private across Korea. There are a total of 40 medical schools in Korea, of which 10 are public and 30 are private schools. Professors in charge of medical education affiliated with the Department of Medical Education were surveyed as medical education experts. Medical school professors with experience in teaching medical students in the fields of basic medicine, clinical medicine, and medical humanities were targeted as participants. The survey recruitment notice were sent to the contact person in the medical education department at each university. All the participants were those who voluntarily participated in the survey online.

### Ethical Approval:

This study was approved by the Gil Medical Center Institutional Review Board of Gachon University (GAIRB2023-448). All participants provided informed consent and participated in the survey after providing consent.

### Measurements:

We created a questionnaire to collect data on the status of ECE programs in Korean medical schools and the educational needs for ECE. The questionnaire was developed according to the Korean medical education context by referring to the survey framework used in previous studies^2^,^8^,^9^ on the status and needs of ECE. To verify the validity of the questionnaire, the contents were revised and supplemented with expert advice from a medical school professor with more than 20 years of teaching experience in medical education and clinical medicine with ECE. This study defined ECE as “early exposure to patients, clinical and medical environments, and hospitals before one begins a clinical clerkship in the medical curriculum” and presented before responding to the survey questions. The questionnaire comprised questions on respondents’ characteristics, the status of ECE programs, and the educational needs for ECE. To determine the status of ECE programs, respondents whose medical schools had ECE programs were asked to provide information about the program, such as the course type, year, semester, and course hours. In Korea, all medical schools follow the same medical education standards regardless of the type of establishment, as they are required by law to meet the evaluation criteria for basic medical education set forth in the accreditation standards of the Korean Institute of Medical Education and Evaluation. In this regard, the survey on ECE programs consisted of the same questions regardless of public and private schools. To determine educational needs, respondents were asked to rate the program’s necessity on a 5-point Likert scale. They were also asked to select responses regarding the objectives and outcomes of ECE programs, the type of education, the teaching methods and assessment methods in ECE programs, and concerns about the implementation of ECE programs.

### Statistical analysis:

The responses were organized using Microsoft Excel 2016 (Microsoft Corp., Redmond, WA, USA). Frequency analysis was used to analyze the status of ECE programs in medical schools and the educational needs for ECE. The frequency analysis was performed using Microsoft Excel 2016 and IBM SPSS version 25.0 (IBM Corp., Armonk, NY, USA).

## RESULTS

Medical education experts and professors from 30 medical schools participated in the survey. Thirty medical education experts filled out the questionnaire on the status of ECE programs. Meanwhile, 30 medical education experts and 65 professors from different medical schools filled out the questionnaire on educational needs. All responses were included in this study. Regarding the characteristics of the participating medical schools, eight (26.7%) were in the metropolitan area and 22 (73.3%) were in the non-metropolitan area. Furthermore, eight (26.7%) were public schools and 22 (73.3%) were private schools. Regarding the representativeness of the sample, it was confirmed that there was no statistically significant difference in the type of establishment and location characteristics of the participating medical schools from the characteristics of medical schools across Korea (*P* < .05). The 65 professors who participated in the survey were affiliated with 11 (16.9%) basic medicine, 45 (69.2%) clinical medicine, 2 (3.1%) medical humanities, 5 (7.7%) medical education, and 2 (3.1%) other. In terms of the professors’ experience in teaching medical students, there were 19 (29.2%) with less than 10 years, 31 (47.7%) with more than 10 years and less than 20 years, 15 (23.1%) with more than 20 years.

### Status of ECE programs in medical schools:

As shown in Table-I, among the 30 participating medical schools, 21 (70.0%) had implemented ECE programs before clinical clerkship in the medical curriculum. Among these 21 medical schools, 10 (47.6%) provided pre-medical phase and 12 (57.1%) provided medical phase. The ECE course was a required course in nine (90.0%) schools providing pre-medical phase and 11 (91.7%) schools providing medical phase. The ECE program was offered in the first year in six (60.0%) schools providing pre-medical phase and nine (75.0%) schools providing medical phase. Regarding the semester, ECE programs were offered in the second semester in seven (70.0%) schools providing pre-medical phase and nine (75.0%) schools providing medical phase. Furthermore, both pre-medical and medical phases of medical schools did not differ based on the number of course hours.

**Table-I:** Details of ECE programs in medical schools (N = 21).

Item	Pre-medical (N = 10)	Medical (N = 12)
***Course type***		
Required	9 (90.0)	11 (91.7)
Elective	2 (20.0)	2 (16.7)
Year		
First	6 (60.0)	9 (75.0)
Second	4 (40.0)	7 (58.3)
***Semester***		
First	3 (30.0)	6 (50.0)
Second	7 (70.0)	9 (75.0)
Summer/Winter vacation	0 (0.0)	1 (8.3)
***Course hours***		
Less than 30 hours	5 (50.0)	6 (50.0)
30 or more hours	5 (50.0)	6 (50.0)

Values are presented as frequency (%).

### Educational needs for ECE in medical schools:

Regarding the need for ECE programs in medical schools, 84 (88.4%) out of 95 respondents agreed that ECE programs are necessary. Regarding the objectives of ECE programs, most respondents (n = 70, 73.7%) selected the option “understanding the role of a doctor,” while the least selected option was “obtaining early education on clinical skills” (n = 16, 16.8%; Table-II). Regarding the outcomes of ECE programs, most respondents (n = 73, 76.8%) selected the option “understanding the role of a doctor.” Less than 10% of respondents selected the options of “development of clinical reasoning” (n = 9, 9.5%), “development of leadership skills” (n = 7, 7.4%), and “development of clinical skills” (n = 1, 1.1%; Table-II).

**Table-II:** Objectives and outcomes of ECE programs in medical schools (N = 95).

Items	N (%)
**Objectives**	
Understanding the role of a doctor	70 (73.7)
Understanding the entire hospital system, including the system of different departments	66 (69.5)
Understanding the role of professionals in other departments	64 (67.4)
Facing and understanding patients’ illness experiences	57 (60.0)
Obtaining early experience in handling clinical situations, including taking patient history and physical examinations and communicating	47 (49.5)
Obtaining experience in community clinical environments	36 (37.9)
Obtaining early education on clinical skills	16 (16.8)
**Outcomes**	
Understanding the role of a doctor (including the responsibility of doctors)	73 (76.8)
Obtaining a holistic understanding of patients and protectors	59 (62.1)
Obtaining motivation to learn about clinical topics	55 (57.9)
Understanding the hospital environment and rescue system	54 (56.8)
Development of system thinking (Understanding the organic interaction system within a hospital surrounding patients)	46 (48.4)
Formation of professional identity	44 (46.3)
Development of collaborative competency	30 (31.6)
Understanding ethics in hospital settings (within medical and hospital departments)	29 (30.5)
Development of communication skills	28 (29.5)
Recognition of the relationship between basic medicine and clinical medicine	18 (18.9)
Development of clinical reasoning	9 (9.5)
Development of leadership skills	7 (7.4)
Development of clinical skills	1 (1.1)

Regarding the type of education in ECE programs, most respondents (n = 61, 64.2%) selected the option “doctor shadowing,” while the least selected option was “patient physical examination” (n = 9, 9.5%; Table-III). In Table-III, most respondents selected “observation/field trips” as a teaching method in ECE programs (n = 69, 72.6%), while the least selected options were “utilizing clinical skill models” (n = 9, 9.5%) and “lecture” (n = 5, 5.3%). Regarding assessment methods in ECE programs, most respondents selected the option “reflection journal” (n = 68, 71.6%). Less than 10% of respondents selected the option of “oral test” (n = 9, 9.5%) and “written test” (n = 3, 3.2%; Table-III). Regarding concerns about the implementation of ECE programs in medical schools, most respondents (n = 68, 71.6%) selected the option “lack of educational support personnel,” while the least selected option was “low learner interests” (n = 12, 12.6%).

**Table-III:** Type of education, teaching methods, and assessment methods in ECE programs in medical schools (N = 95).

Items	N (%)
***Type of education***	
Doctor shadowing	61 (64.2)
Visit to community medical facilities (such as national medical center, public healthcare, and primary care)	40 (42.1)
Observation of outpatient treatment	36 (37.9)
Observation of administrative departments within hospitals	35 (36.8)
Inpatient care	34 (35.8)
Nurse shadowing	25 (26.3)
Interview with patients and protectors	24 (25.3)
Observation of examination rooms and operating rooms	22 (23.2)
Taking patient history	20 (21.1)
Clinical skills education	14 (14.7)
Patient physical examination	9 (9.5)
***Teaching methods***	
Observation/Field trips	69 (72.6)
Small group learning (such as case-, problem-, and team-based learning)	59 (62.1)
Utilizing real patients	39 (41.1)
Role-play	31 (32.6)
Utilizing simulated patients	27 (28.4)
Presentation	19 (20.0)
Utilizing virtual patients	17 (17.9)
Writing reports	14 (14.7)
Utilizing clinical skill models	9 (9.5)
Lecture	5 (5.3)
***Assessment methods***	
Reflection journal	68 (71.6)
Portfolio	42 (44.2)
On-site instructor assessment (checklist)	35 (36.8)
Presentation	33 (34.7)
Peer assessment	26 (27.4)
Self-assessment	24 (25.3)
Report	23 (24.2)
Clinical Performance Examination	17 (17.9)
Objective Structured Clinical Examination	12 (12.6)
Attendance	12 (12.6)
Oral test	9 (9.5)
Written test	3 (3.2)

## DISCUSSION

In Korea, studies on ECE have been conducted on the experience analysis in a single medical school.^15^,^16^ However, no research has been conducted to analyze the status of ECE programs in Korean medical schools nationwide. In this regard, this study is significant in identifying the status of the programs in medical schools across Korea and the educational needs for ECE to determine directions for implementing ECE programs in Korea’s undergraduate medical education. In addition, the findings of this study provide useful information for understanding cross-country differences in ECE education through comparisons with previous studies from other countries.

A previous study investigated the status of preclinical preparation education in Korea and found that 92.7% of medical schools offer such courses mainly in the medical phase, particularly from the second semester of the second year to the first semester of the third year.^17^ They also reported that the courses are more likely prepared for clinical clerkships than for ECE. In contrast, the respondents of this study answered questions about programs that conform to the concept of ECE. This study confirmed that 70.0% of Korean medical schools offer ECE programs from the first year of pre-medical phase to the second year of medical phase. Therefore, it can be confirmed that more than half of Korean medical schools have implemented ECE programs apart from preclinical preparation education. This study also found that 88.4% of respondents agreed with the need for ECE programs, confirming the high awareness of the programs in Korean medical education.

Başak et al., who examined the status of ECE in European medical schools and found that many people consider obtaining clinical and communication skills as essential ECE objectives.^8^ In contrast, the respondents of this study considered “understanding the role of a doctor” an essential educational objective of ECE programs in Korean medical schools. Our results also showed that “utilizing clinical skill models” is not commonly considered an essential ECE teaching method in Korea, whereas European medical schools frequently utilize these skills.^8^ Considering that perceptions of essential ECE educational objectives and teaching methods differ between Korea and Europe, it suggests the need to define the concept of ECE and develop a curriculum suitable for Korean medical education.

Medical schools in Europe have reported that difficulty allocating time in the curriculum hinders Early Clinical Exposure (ECE), citing reasons such as an overloaded curriculum, resistance from senior faculty, and the absence of a dedicated department to coordinate ECE programs.^9^ Unlike these results, many respondents reported the “lack of educational support personnel” and “difficulty cooperating or coordinating with hospitals” as concerns about the implementation of ECE programs in Korean medical schools. However, the next most significant obstacle in European medical schools is faculty-related difficulties.^9^ This result aligns with the result of this study, as the next most common concern was “difficulties in faculty development.” This result shows the importance of faculty development and faculty participation in the successful implementation of ECE programs. Therefore, it is necessary to develop curricula and teaching methods and promote faculty participation, support, and cooperation at the institutional level.

Medical schools are aware of the crucial need for ECE programs, but research on this topic still needs improvement in Korea. ECE must be recognized as a means to realize vertical integration of knowledge between basic and clinical medicine and foster competent doctors. Research on the educational effect of ECE in medical education continues to be published,^10^-^14^,^18^,^19^ and in India, ECE guidelines have been established for undergraduate medical education.^20^ In addition, this study comprehensively examined the perceptions of medical education experts and professors about the educational needs for ECE. However, future studies should examine these educational needs among medical students. After identifying learners’ needs, the results can be compared with the results of this study, paving the way for more effective educational programs.

### Limitations:

This study analyzed the responses of those individuals who voluntarily participated in this study. Out of the 40 medical schools targeted, 10 did not participate in this study.

## CONCLUSION

This study confirmed that ECE programs in medical schools across Korea are mainly offered as required courses in the pre-medical and medical phases before clinical clerkship. In addition, it was derived from the professors’ perspectives that a high awareness of the necessity of ECE programs and specific matters to be considered for implementing ECE programs in the future. The results can serve as foundational data for designing future ECE programs, thereby shaping the future of medical education and realizing vertical integration education in Korea. Furthermore, the results can provide valuable insights into the status of ECE programs and the educational needs of ECE in the context of Korean as well as Asian medical education.

### Authors’ Contributions:

**SK SYK::** Contributed to the data curation, methodology, formal analysis, validation and the original draft writing. **KHP:** Contributed to the study concept, project administration, data curation, methodology, formal analysis, validation and the original draft writing. **SJY:** Contributed to the study concept, funding acquisition. All authors have approved the final version and are responsible and accountable for the accuracy and integrity of the work.
